# Maternal thyroid function and the outcome of external cephalic version: a prospective cohort study

**DOI:** 10.1186/1471-2393-11-10

**Published:** 2011-01-26

**Authors:** Simone M Kuppens, Libbe Kooistra, Tom H Hasaart, Riet W van der Donk, Huib L Vader, Guid S Oei, Victor J Pop

**Affiliations:** 1 Department of Primary Care, University of Tilburg, Tilburg, the Netherlands; 2 Department of Obstetrics and Gynecology, Catharina Hospital, Eindhoven, the Netherlands; 3 Department of Pediatrics, Alberta Children's Hospital, University of Calgary, Canada; 4 Department of Clinical Chemistry, Máxima Medical Centre, Veldhoven, the Netherlands; 5 Department of Biomedical Engineering, University of Technology, Eindhoven, the Netherlands; 6 Department of Obstetrics and Gynecology, Máxima Medical Centre, Veldhoven, the Netherlands; 7 Department of Electrical Engineering, University of Technology, Eindhoven, the Netherlands

## Abstract

**Background:**

To investigate the relation between maternal thyroid function and the outcome of external cephalic version (ECV) in breech presentation.

**Methods:**

Prospective cohort study in 141 women (≥ 35 weeks gestation) with a singleton fetus in breech. Blood samples for assessing thyroid function were taken prior to ECV. Main outcome measure was the relation between maternal thyroid function and ECV outcome indicated by post ECV ultrasound.

**Results:**

ECV success rate was 77/141 (55%), 41/48 (85%) in multipara and 36/93 (39%) in primipara. Women with a failed ECV attempt had significantly higher TSH concentrations than women with a successful ECV (p < 0.001). Multiple logistic regression showed that TSH (OR: 0.52, 95% CI: 0.30-0.90), nulliparity (OR: 0.11, 95% CI: 0.03-0.36), frank breech (OR: 0.30, 95% CI: 0.10-0.93) and placenta anterior (OR: 0.31, 95% CI: 0.11-0.85) were independently related to ECV success.

**Conclusions:**

Higher TSH levels increase the risk of ECV failure.

**Trial registration number:**

ClinicalTrials.gov: NCT00516555

## Background

The incidence of breech presentation decreases with increasing gestational age from approximately 16% at 32 weeks to 3-5% of all pregnancies at term [1]. External cephalic version (ECV) is recommended by the American College of Obstetricians and Gynecologists (ACOG) and the Royal College of Obstetricians and Gynaecologists (RCOG) as the best method to reduce the number of breech presentations and breech deliveries at term [2,3]. Also the Dutch Obstetrics & Gynaecology Society has promoted ECV but up until now, the implementation of this guideline is rather poor and a recent study has been started to evaluate possible barriers [4]. Reported success rates vary widely from 30-80% [3]. Several factors have been associated with successful ECV outcome including parity, type of breech, placenta location, amniotic fluid index, and engagement of the breech [5-7].

Recently, our research group showed that at 36 weeks gestation women with TSH levels above 2.5 mIU/l are at risk for breech presentation [8,9]. It is stated that fetal movements and mobility are a prerequisite for spontaneous version into the cephalic position [10]. Since maternal thyroid function is related to the probability of spontaneous cephalic presentation, it may be hypothesized that TSH may, also, affect ECV outcome [8,9].

To the best of our knowledge, this is the first prospective study to examine the relationship between maternal thyroid hormone status and ECV success.

## Methods

### Participants

Over a period of two years, 194 women were prospectively seen in a specialized ECV outpatient clinic and invited to participate when they presented at ≥ 35 weeks gestation with a fetus in breech presentation. Only Caucasian patients with sufficient knowledge of the Dutch language were eligible (n = 169). Nineteen patients declined to participate, while four patients with a transverse lie and five patients with known thyroid disease were excluded. Data analysis, therefore, included the remaining 141 participants (see Table 1), all of whom gave written informed consent.

**Table 1:** Characteristics and thyroid parameters of 141 breech women who had an ECV attempt.

	Mean (SD)	N (%)
**Demographic features**		

Maternal age (years)	31.6 (4.2)	
Range (23 - 46)		

Any tobacco use		11 (8)

Any alcohol use		12 (8.5)

Body mass index (BMI)		
Range (17 - 47)	23.7 (4.2)	

BMI < 25		112 (79.5)

BMI between 25 and 30		17 (12)

BMI > 30		12 (8.5)

Previous miscarriage in life		31 (22)

**Obstetrical features**		

Nulliparity		93 (66)

Multiparity		48 (34)

Gestational age at ECV (wks)	36.1 (0.9)	
Range (35.0-39.9)		

*Type of breech*		

Frank		93 (66)

Complete		36 (25.5)

Incomplete		12 (8.5)

*Placenta location*		

Anterior		55 (39)

Posterior		56 (40)

Lateral		30 (21)

Cephalic presentation after ECV		77 (55)

Cephalic presentation at delivery		79 (56)

Placental weight (grams)	615 (127)	
Range (295 - 1000)		

Birth weight (grams)	3344 (447)	
Range (1765 - 4576)		

**Thyroid parameters**		

TSH mIU/l		
median	1.48	
Range (0.02 - 10.0)		

FT4 pmol/l		
median	10.0	
Range (7.1 - 14.0)		

TPO-Ab > 35 kU/l		9 (6)

This study was approved by the Medical Ethical Committee of the Catharina Hospital in Eindhoven, the Netherlands.

### Assessments

At intake, demographic features (maternal age, BMI, tobacco/alcohol use) and obstetrical parameters (parity, previous obstetrical history) were recorded. Prior to ECV, ultrasound was performed to determine type of breech, amniotic fluid index and placental location. Ultrasound was repeated post-ECV in order to confirm success. At birth, fetal presentation, placental and birth weight were carefully assessed.

### ECV intervention

Cardiotocography was performed routinely before and after the procedure. Before ECV, all women had a full bladder and were routinely given a tocolytic drug (Atosiban, 6.75 mg. intravenously). Anti-D (1000 IE) was given prophylactically to all Rhesus negative women. ECV (monitored by ongoing ultrasound) was performed by two experienced obstetricians working in unison: the hands of one staff member concentrated on the breech, while the other's concentrated on the fetal head, with manipulation being consecutive rather than simultaneous (see Additional file 1). While a "forward somersault" was the preferred method to achieve cephalic position, a "backward flip" was an alternative strategy for nulliparous patients with a frank breech presentation. ECV was defined as successful when cephalic position was demonstrated on the post-ECV ultrasound.

### Thyroid parameters

Blood samples were taken prior to administration of the tocolytic drug and subsequently stored for assessing the following thyroid parameters: thyroxine (FT4), thyrotrophin (TSH) and thyroid peroxidase antibodies (TPO-Ab). TSH was measured using a solid-phase, two-site chemiluminescent enzyme immunometric assay (IMMULITE Third generation TSH, Diagnostic Products Corporation, Los Angeles USA). The inter-assay coefficients of variation were 5.0% and 4.4% at concentrations 0.22 mIU/l and 2.9 mIU/l, respectively. FT4 concentration was also measured by means of a solid-phase immunometric assay (IMMULITE Free T4). The inter-assay coefficients of variation for this technique were 6.7% and 4.4% at concentrations of 11.6 pmol/l and 31.5 pmol/l, respectively. Reference ranges for non-pregnant women for TSH and FT4 were: 0.4 - 4.0 mIU/l and 10 - 24 pmol/l, respectively. The IMMULITE TPO-Ab kit was used for the determination of antibodies against thyroid peroxidase (TPO). The inter-assay coefficients of variation for this analysis were 9% and 9.5% for concentrations of 40 kU/l and 526 kU/l, respectively. The anti-TPO assay was standardized in terms of the International Reference Preparation for anti-TPO MRC 66/387. TPO-Ab concentrations > 35 kU/l at 36 weeks gestation were regarded as antibody positive.

### Statistical analysis

Statistical analysis was performed using the Statistical Package for the Social Sciences (SPSS 17.0). Initial analyses described the sample using means, standard deviations and frequencies. The distributions of FT4 and TSH were non-Gaussian (Kolmogorov-Smirnov tests); therefore, Mann-Whitney U-tests were used to examine FT4 and TSH for group comparisons and ECV success. Associations among elevated maternal thyroid antibodies (TPO-Ab > 35 kU/l) and ECV outcome were analyzed using chi-square tests. Prevalence of successful ECV stratified according to quartiles of TSH was also examined using a chi-square test. Single and multiple logistic regression analysis (odds ratios and 95% CI's) were performed to calculate un-adjusted and adjusted ORs for successful ECV (dependent variable). The independent variable was TSH. For the multiple logistic regression analysis, age (continuous), body mass index (continuous), parity (dichotomized in nulliparous and parous), type of breech (frank breech versus the rest), gestational age at version (continuous), placental position (dichotomized in anterior versus other), placental weight (continuous), birth weight (continuous) and amniotic fluid index (dichotomized using 10 cm as a cutoff) were also controlled for.

## Results

The overall ECV success rate, indicated by the post-procedure ultrasound results, was 55% (77/141), and was significantly lower in nulliparous women (39%: 36/93) than in multiparous women (85%: 41/48; χ^2 ^= 27, df = 1, p < 0.001). In one patient prolonged post-ECV fetal bradycardia occurred which necessitated emergency cesarean section. Transient heart rate abnormalities were observed in 3 patients. Spontaneous version into cephalic position at birth after failed ECV occurred in 2 (nulliparous) women. Spontaneous version back into breech after successful ECV did not occur.

The median TSH level for the whole study sample was 1.48 mIU/l (range: 0.02 - 10 mIU/l). Table 2 shows the distribution of FT4 and TSH values in relation to ECV outcome. Women with failed ECV's had significantly higher TSH levels than those with successful ECV's (Mann-Whitney U, p < 0.001), but corresponding FT4 levels did not differ between groups (Mann-Whitney U, p = 0.88). The correlation between FT4 and TSH was 0.18, (p = 0.034). Also, the prevalence of elevated TPO-Ab (> 35 kU/l) did not differ between groups (9% versus 4%, p = 0.18) with regard to ECV outcome.

**Table 2:** Thyroid parameters in 141 breech women in relation to ECV outcome

	Success ECV N = 77	Failed ECV N = 64	P Mann- Whitney U	P χ^2 ^(df = 1)
Median TSH mIU/l	1.20	1.90	< 0.001	
Range	0.02-10.0	0.60-9.20		

Median FT4 pmol/l	10.0	10.0	0.88	
Range	7.2-14.0	7.1-14.2		

TPO-Ab > 35 kU/l	3 (4%)	6 (9%)		0.18

**Figure 1:**
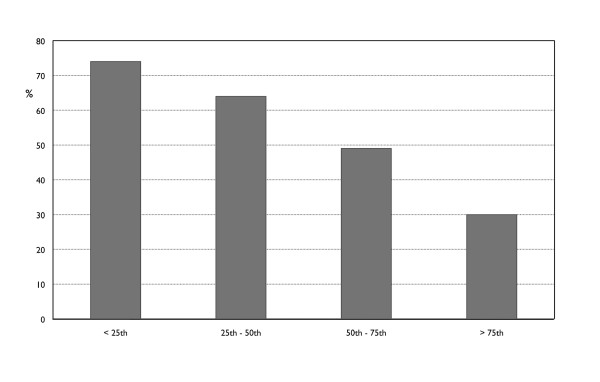
**Percentage of ECV success in relation to TSH quartiles (mIU/l) in 141 women with breech presentation ≥ 35 weeks gestation**.

Figure 1 shows the prevalence of successful ECV according to different TSH quartiles (25^th ^percentile: < 0.99 mIU/l, 25^th ^- 50^th ^percentile: 0.99 - 1.48 mIU/l, 50^th ^- 75^th ^percentile: 1.49 - 2.0 mIU/l and > 75^th ^percentile: > 2.0 mIU/l). Clearly, the percentage of ECV success significantly decreased with increasing TSH quartile (74% to 30%, χ^2 ^= 15, df = 3, p = 0.002).

In Table 3, single logistic regression is shown with successful ECV as the dependent variable. TSH, parity, type of breech and placenta anterior location were all significantly related to successful ECV outcome. In Table 3, adjusted OR's are shown with TSH as independent variable and successful ECV as dependent variable using multiple logistic regression analysis. TSH (OR: 0.52, 95% CI: 0.30-0.90), nulliparity (OR: 0.11, 95% CI: 0.03-0.36), frank breech (OR: 0.30, 95% CI: 0.10-0.93) and placenta anterior (OR: 0.31, 95% CI: 0.11-0.85) were all significantly and independently related to ECV success.

**Table 3:** Logistic regression analysis (dependent variable: successful outcome of ECV)

	O.R.	95% C.I.
*Unadjusted odds ratios*		

Maternal age	1.05	0.97-1.14

Body Mass Index	0.93	0.86-1.02

Gestation at version	1.05	0.73-1.50

**Nulliparity**	**0.12**	**0.05-0.29**

**Frank breech**	**0.37**	**0.18-0.78**

Amniotic fluid > 10 cm	1.33	0.67-2.65

**Placenta anterior**	**0.36**	**0.18-0.74**

Placental weight	1.00	0.99-1.01

Birth weight	1.001	1.00-1.002

**TSH**	**0.59**	**0.41-0.88**

FT4	1.03	0.83-1.29

*Adjusted odds ratios*		

Maternal age	1.02	0.91-1.14

Body Mass Index	0.88	0.76-1.01

Gestation at version	0.91	0.53-1.56

**Nulliparity**	**0.11**	**0.03-0.36**

**Frank breech**	**0.30**	**0.10-0.93**

Amniotic fluid index > 10	1.21	0.51-2.34

**Placenta anterior**	**0.31**	**0.11-0.85**

Placental weight	0.99	0.98-1.01

Birth weight	1.001	1.000-1.002

**TSH**	**0.52**	**0.30-0.90**

FT4	1.15	0.82-1.61

In healthy pregnant women (without elevated concentrations of TPO-Ab and with proven sufficient iodine intake) trimester specific cutoffs for elevated TSH are still lacking. A frequently used upper TSH reference level in pregnancy is ≥ 2.5 mIU/l [11]. In the current study 20 women had a TSH above this level. Of these 20 women 5 (25%) underwent successful ECV, compared to 72 successful ECVs from the sample of 121 women (60%) with TSH levels below the cutoff of 2.5 mIU/l (χ^2 ^= 8.2, df = 1, p = 0.004). The RR of women in breech with TSH above 2.5 mIU/l for failed ECV was 2.4, (95% CI = 1.1 - 5.3).

## Discussion

In the current study, successful ECV outcome was independently related to maternal TSH: women with unsuccessful ECV had significantly higher TSH levels.

The ECV success rate of 55% in the current study is comparable to others reported in the literature [2,3]. While our data support earlier observations showing that factors affecting ECV success include parity, type of breech and placenta location, to our knowledge, this is the first study reporting on a possible relationship between maternal thyroid function and ECV outcome [5-7].

We recently showed in a large sample of over 1000 pregnant women from the general population that higher levels of TSH constitutes a risk factor for breech presentation [8].

How can the observed relationship between maternal TSH and ECV outcome be explained? Several mechanisms might be suggested. First, one could argue that elevated maternal TSH directly impacts uterine relaxation, thereby negatively affecting ECV outcome. Empirical evidence regarding such a direct effect is, however, scarce. TSH receptors have been shown in other non-thyroid tissues such as bone, heart and brain tissue, but so far no studies on human uterine samples have been published [12,13]. Also, in patients with subclinical hypothyroidism (reflected by high TSH), the myocytes of the smooth muscle cells of the aorta are dysfunctional, leading to increased arterial stiffness and impaired diastolic function [14]. The mechanism behind is a reduced activity of the sarcoplasmatic reticulum CA-ATPase which controls the efficient concentration of calcium in the cytoplasm. Another study in sub-clinical hypothyroid women showed a beneficial effect of thyroxine replacement on arterial stiffness [15]. It is known from earlier studies that thyroid hormone (T3) has a direct relaxation effect on vascular smooth muscle cells [16]. It might be hypothesized that a similar relation between TSH and uterine myocytes does exist. Inappropriate relaxation of uterine smooth muscle vessels in women with high TSH might hinder ECV. Another candidate mechanism might relate to fetal motility and its ability to facilitate spontaneous version. High maternal TSH during gestation has been associated with impaired neonatal motor function [17]. One may argue that higher levels of maternal TSH could conceivably reflect low circulating fetal T4 levels. Shortage of fetal T4 compromises fetal neural maturation and, in turn, fetal motility through its effects on muscle tone and reflexes [18]. Furthermore, breech babies tend to be smaller, have lower scores on neuromotor tests, and are balanced-impaired until the age of 12-18 months [19-22]. Their impaired neuromotor development could in fact hinder ECV outcome: it might be suggested that poor fetal movements result in less expanded intra-uterine environment which is needed for successful ECV.

The current study findings did not reveal differences in FT4 level between the successful versus the unsuccessful ECV groups. This may have been due to the greater sensitivity of TSH - compared to FT4 - to detect small changes in the set point of the hypothalamus-pituitary-thyroid axis [23]. Moreover, it has been reported that pregnancy FT4 assessments are less reliable than non-pregnancy assessments [24]. Also in this study a weak correlation (r = 0.18, p = 0.34) was found between FT4 and TSH, but in the opposite direction of what would be expected: normally there is an inverse correlation between TSH and FT4.

Despite growing insight into the prognostic factors of successful ECV, no scoring system that accurately predicts success exists [25-28]. It may well be that the inclusion of maternal TSH would lead to enhanced probability-of-ECV success estimates.

A limitation of the current study relates to the fact that iodine excretion data in mothers and neonates were not assessed. This made it impossible to relate higher TSH levels to inadequate iodine intake during gestation. Also cord blood was not assessed and therefore fetal TSH and T4 levels could not be taken into account.

A major strength of the current study was its relatively large sample size and the fact that ECV was performed in one obstetric department by a small team of trained experts following the same protocol and blind to the thyroid status of the patients.

## Conclusions

In conclusion, the current study is among the first to report a possible relationship between maternal thyroid function and ECV outcome. While the study suggests that higher levels of TSH are independently related to ECV outcome, the precise mechanism behind this finding remains to be determined.

## Competing interests

The authors declare that they have no competing interests.

## Authors' contributions

SK, RD, TH and VP were involved in conception and design of the study.

SK, HV and VP analysed the data.

SK, LK, TH and VP drafted the first manuscript.

GO, HV and RD contributed to data analysis and interpretation.

All authors read and approved the final manuscript.

## Pre-publication history

The pre-publication history for this paper can be accessed here:

http://www.biomedcentral.com/1471-2393/11/10/prepub

## Supplementary Material

Additional file 1 **ECV technique**. ECV is performed by two experienced obstetricians working closely thogether: the hands of one obstetrician concentrate on the breech, while the other's concentrate on the fetal head, with manipulation being consecutive rather than simultaneous and changing from a "pull"movement into a "push"movement. The procedure is monitored by a third person by ongoing ultrasound. Click here for file
