# Supplementary material for: Maternal thyroid function and the outcome of external cephalic version: a prospective cohort study
Source: BMC Pregnancy Childbirth. 2011 Jan 26;11:10. doi: 10.1186/1471-2393-11-10 (PMC3042424; doi:10.1186/1471-2393-11-10)
Supplement: Additional file 1 — ECV technique. ECV is performed by two experienced obstetricians working closely thogether: the hands of one obstetrician concentrate on the breech, while the other's concentrate on the fetal head, with manipulation being consecutive rather than simultaneous and changing from a "pull"movement into a "push"movement. The procedure is monitored by a third person by ongoing ultrasound. [file 1471-2393-11-10-S1.ZIP › index.html]

Succesful External Cephalic Version in Breech


 
